# A Novel Function of TET2 in CNS: Sustaining Neuronal Survival

**DOI:** 10.3390/ijms160921846

**Published:** 2015-09-10

**Authors:** Yajing Mi, Xingchun Gao, Jinxiang Dai, Yue Ma, Lixian Xu, Weilin Jin

**Affiliations:** 1 State Key Laboratory of Military Stomatology, Department of Anesthesiology, School of Stomatology, the Fourth Military Medical University, Xi’an 710032, China; E-Mail: miyajing@163.com; 2 Institute of Basic Medicine Science, Xi’an Medical University, Xi’an 710021, China; E-Mail: gxc199281003@163.com; 3 School of Life Sciences and Biotechnology, Shanghai Jiao Tong University, Shanghai 200240, China; 4 Department of Cell and Developmental Biology, University of Colorado Denver, Denver, CO 80045, USA; E-Mail: jin.dai@ucdenver.edu; 5 Institute of Nano Biomedicine and Engineering, Department of Instrument Science and Engineering, Key Laboratory for Thin Film and Microfabrication Technology of Ministry of Education, School of Electronic Information and Electronic Engineering, Shanghai Jiao Tong University, Shanghai 200240, China; E-Mail: mayuels@126.com

**Keywords:** TET2, cell survival, CNS, neurons, RNA interference

## Abstract

DNA dioxygenases Ten-Eleven Translocation (TET) proteins can catalyze the conversion of 5-methylcytosine (5mC) of DNA to 5-hydroxymethylcytosine (5hmC), and thereby alter the epigenetic state of DNA. The TET family includes TET1, TET2 and TET3 members in mammals. Recently, accumulative research uncovered that TET1–3 occur abundantly in the central nervous system (CNS), and their biological functions have just begun to be investigated. In the present study, we demonstrated that mRNA and protein of TET2 were highly expressed in the cerebral cortex and hippocampus along the whole brain-development process. Further studies showed that TET2 was expressed in various types of cells, especially in most neurons. Subcellular distribution pattern implicated that TET2 is localized in both nucleus and cytoplasm of neurons. Down-regulation of TET2 in cultured cortical neurons with RNA interference implied that TET2 was required for cell survival. In all, our results indicate that neuronal TET2 is positively involved in the regulation of cell survival.

## 1. Introduction

DNA methylation is one of the best-characterized epigenetic modifications. Recent studies have indicated that the Ten-Eleven Translocation (TET) proteins could catalyze the conversion of 5-methylcytosine (5mC) of DNA to 5-hydroxymethylcytosine (5hmC), which may mediate DNA demethylcytosine [1,2]. The mammalian TET family includes three members, TET1, TET2, and TET3, all of which share a high degree of homology within their C-terminal catalytic domains [3]. TET1 is indispensable for maintaining embryonic stem (ES) pluripotency and inner cell mass cell specifications [2,4]. Disruption or deletion of TET2 function could impair hematopoietic cell homeostasis and hematopoietic differentiation, and subsequently led to the development of myeloid malignancies [5,6,7]. TET3-mediated DNA hydroxylation is involved in the epigenetic reprogramming of zygotic paternal DNA following natural fertilization, and may also contribute to somatic cell nuclear reprogramming during animal cloning [8].

Recently, 5hmC and TET1–3 have been reported to be abundant in the brain [2,9,10,11], especially TET2, and the expression level is steadily sustained along central nervous system (CNS) development [11,12]. However their potential functions are largely unknown. Consistently, high levels of 5hmC are, not only present in neuronal progenitors, but can also be seen in post-mitotic neurons [13]. Furthermore, the 5hmC value rises during the neuronal differentiation process and is enriched in activated neuronal function-related genes [14]. Mice lacking TET1 exhibited impaired neurogenesis of the hippocampus, accompanied by poor learning and memory [15]. Up-regulation of TET1 was found in the parietal cortex of psychotic patients [16]. Functional perturbation of TET2 and TET3 led to defects in neuronal differentiation [14]. Recent research further suggested that TET3 played essential roles in neural progenitor cell maintenance, terminal differentiation, and rapid behavioral adaptation [17,18].

In this report, we found that mRNA and protein of TET2 were expressed abundantly in the cortex and hippocampus. Furthermore, TET2 was largely distributed in most neurons, including cytoplasm, nucleus, and neurites. Importantly, functional experiments showed that primary cortical neurons depleted of TET2 were more prone to die. These data suggest that neuronal-distributed TET2 possibly exerts an essential role on cell survival, and the molecular mechanism should be further studied.

## 2. Results and Discussion

### 2.1. Spatio-Temporal Expression Pattern of Ten-Eleven Translocation 2 (TET2) in Central Nervous System (CNS)

**Figure 1:**
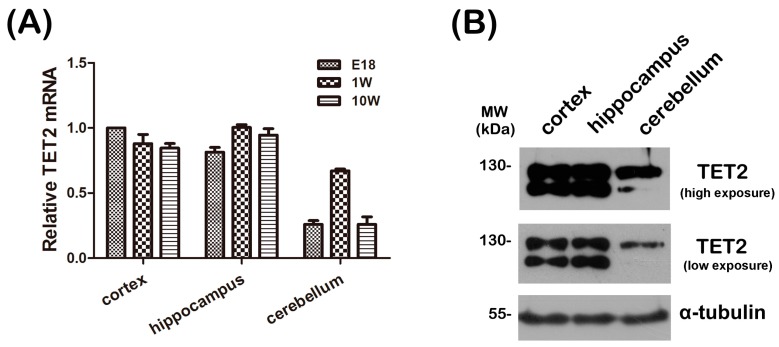
(**A**) The total RNA was extracted from cerebral cortex, hippocampus and cerebellum of E18, 1-week and 10-week mice, respectively, and then subjected to quantitative real-time PCR. β-Actin was selected as an inner standard. TET2 mRNA of E18 cortex was represented as unit 1; (**B**) 30 μg total protein of cerebral cortex, hippocampus, and cerebellum from 10-week mice were extracted and subjected to Western blot with TET2, α-tubulin was selected as a loading control.

To explore the expression level of TET2 in the CNS, real-time PCR was first performed to investigate the spatio-temporal expression pattern of TET2. The total RNA of cerebral cortex, hippocampus, and cerebellum of Embryonic 16–18 day (E18), one-week, and 10-week mice were extracted, respectively, and mRNA levels of TET2 were examined. Quantitative analysis implicated that the TET2 mRNA level was relatively higher in the cerebral cortex and hippocampus tissues, and is stably sustained during the whole development of mice brain (Figure 1A). Next, the total protein of cerebral cortex, hippocampus, and cerebellum of adult mice were extracted and subjected to Western blot with anti-TET2 antibody. Data showed that TET2 protein was largely expressed in the cerebral cortex and hippocampus, but slightly expressed in cerebellum (Figure 1B). Notably, there were two bands around 130 kDa in the three tissues, the lower band might be the degraded fragments of TET2 protein (Figure 1B). These two consistent results clearly informed us that mRNA and protein of TET2 were very stable in the entire development process of the CNS, especially in the cerebral cortex and hippocampus.

### 2.2. Intraneuronal Distribution of TET2

**Figure 2:**
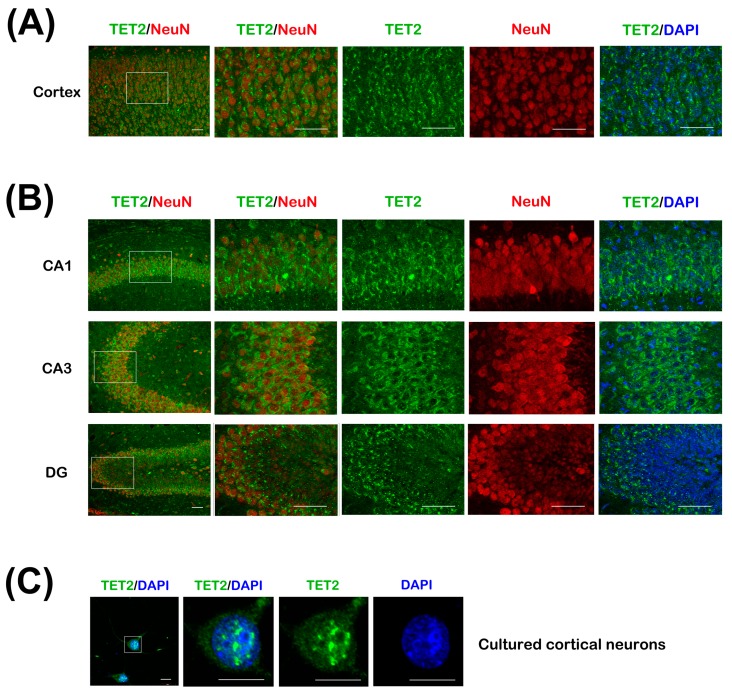
(**A**) The cerebral cortex sections of 8-week mice were co-stained with TET2 and NeuN antibodies, DAPI was stained for nuclear. The white rectangle region of upper cortex in the left image was magnified and showed on the right four panels. Bar = 50 μm; (**B**) CA1, CA3, and dentate gyrus (DG) regions of hippocampus sections were co-stained as in (**A**). Bar = 50 μm; (**C**) Primary cortical neurons at 3 days *in vitro* (DIV) were stained with TET2 antibody and DAPI. The neuron in the white rectangle region of the left image was magnified and showed on the right panels. Bar = 10 μm.

To further clarify the cellular distribution of TET2 in the cerebral cortex and hippocampus, immunohischemical staining was performed with anti-TET2 and anti-Neuronal Nucleus (NeuN) antibodies, NeuN is a neuronal-specific maker. The low magnification of cross sections of the upper cortex in adult mice showed that TET2 positive staining signals could be clearly detected in most neurons and a few other cells, which were possibly oligodendrocytes and astrocytes (Figure 2A). This was further illustrated for the region shown at a higher magnification. In these neurons, with the co-staining of 4,6-diamino-2-phenyl indole (DAPI), we found that TET2 was mainly distributed in the cytoplasm and less in nucleus. In CA1, CA3, and dentate gyrus (DG) regions of hippocampus, TET2 distribution exhibited similar expression patterns as in the upper cortex (Figure 2B).

To further confirm the subcellular distribution of TET2 in neurons, we performed immunostaining for TET2 in primary cultured cortical neurons at three days *in vitro* (DIV). Unlike the tissue sections, confocal scanning images demonstrated a dispersed appearance in the cytoplasm and neurites, and strong immunoreactivity in the nucleus (Figure 2C). Focusing on the nucleus at magnified images, TET2 protein appeared as coarse granulars. Double labeling with DAPI indicated that the granular immunity was among the weak DAPI area, which might well be the euchromatic region (Figure 2C).

### 2.3. Neuronal Distributed TET2 Plays an Important Role in Cell Survival

**Figure 3:**
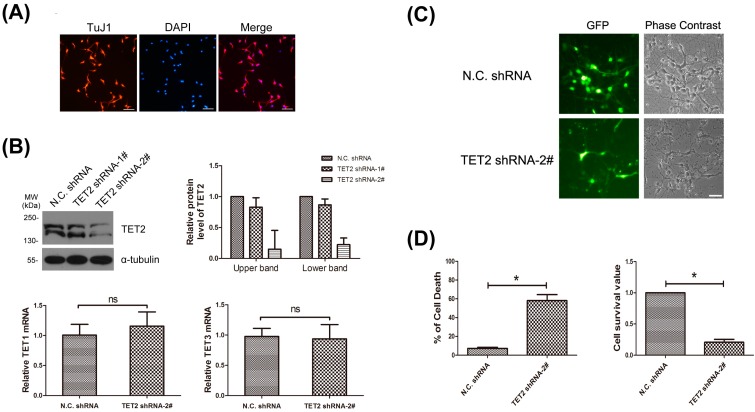
(**A**) Primary neurons were marked with TuJ1 antibody, and DAPI was stained for nuclear of all cells. Bar = 50 μm; (**B**) Primary neurons were infected with lentiviruses carrying N.C. shRNA, TET2 shRNA-1# and TET2 shRNA-2# respectively. Seventy-two hours later, total protein of the three groups of neurons were extracted and blotted with TET2 antibody, α-tubulin was selected as a loading control. The expression level of TET2 protein in each group was quantified with IPP6.0 software in three independent experiments (**upper**). Meantime, the total RNA of neurons from N.C. shRNA group and TET2 shRNA-2# group were extracted, and then TET1 and TET3 mRNA level was exmined with quantitativereal-time PCR (**lower**). *n* = 3, Mean ± S.D., paired *t*-test, ns, not significant; (**C**) Primary neurons were infected with N.C. shRNA and the effective TET2 shRNA-2# respectively after plating, both of which were tagged with GFP, then fluorescence and phase contrast images were collected under a Leica microscope 72 h later. Bar = 50 μm; (**D**) As in (**C**), 72-h later, neurons were double-stained with PI and Hoechst, then cell death rate was examined by PI (+)/Hoechst (+). *n* = 3, Mean ± S.D., paired *t*-test, * *p* < 0.05 (**left**). Using another survival rate test, neurons were incubated with MTT for 4 h, and then the cell survival value was analyzed. Cell survival value of N.C. shRNA was represented as unit 1. *n* = 3, Mean ± S.D., paired *t*-test, * *p* < 0.05 (**right**).

To determine the potential functions of neuronal TET2, primary cortical neurons were used. The purity of neurons was tested with staining of TuJ1 (class III β-tubulin, another neuron-specific marker) and DAPI, and it reached more than 90% (Figure 3A). Two lentivirus-mediated shRNA against TET2 were designed and synthesized. The efficacy of the shRNAs was checked in primary neurons. Western blot data showed that the protein level of endogenous TET2 was markedly down-regulated using the TET2 shRNA-2# (Figure 3B, upper). Meanwhile, endogenous TET1 and TET3 expression did not change with quantitative real-time PCR (Figure 3B, lower).

Primary cortical neurons were infected with lentivirus-mediated TET2 shRNA-2#, which was tagged with a green fluorescent protein (GFP) reporter. Seventy-two hours later, 90%–95% of the neurons were GFP positive under a fluorescence microscope, indicating that almost all neurons were successfully infected (Figure 3C, left panels). Meanwhile, we found that a large number of neurons in TET2 shRNA-2# group were more likely to die compared with the N.C. shRNA group, including disintegrated cell bodies and degenerative neurites (Figure 3C, right panels). Then, the cell death rate was analyzed by a double-staining of PI and Hoechst, the N.C. shRNA group was 7.23%, while TET2 shRNA group was 58.13% (Figure 3D, left panel). Consistently, MTT assay showed that, when TET2 was down-regulated, cortical neurons were unable to maintain normal phenotype and the cell survival value was only 23.55% of the N.C. shRNA group (Figure 3D, right panel). All these data showed that neuronally-expressed TET2 was necessary for cell survival, and the mechanism should be further studied.

### 2.4. Discussion

Previous studies indicated TET1–3 were abundant in the CNS using Western blot and real-time PCR, and TET2 expression was higher [11,12]. To clarify TET2 distribution patterns in neurons, we performed double staining by NeuN and TET2 on brain sections. Data showed that TET2 was expressed in most neurons, including cortical neurons and hippocampal neurons (Figure 2A,B). Meanwhile, *in vivo* and *in vitro* subcellular expression patterns of neurons implied that TET2 could be detected in both nucleus and cytoplasm (Figure 2A–C). These results were consistent with the Western blotting data for subcellular detection of TET2 in mice brain in our previously published article [19]. Nuclear TET2 mainly localized in the euchromatic region, which represented the active gene transcription area (Figure 2C). In addition to nucleus, TET2 could be also found in the cytoplasm, especially in the cortex, CA1, CA3, and DG regions (Figure 2A,B). Some studies also have confirmed TET2 distribution in both mitochondria and nucleus [11,20,21]. In the present study, two bands around 130 kDa could be detected with TET2 blotting in brain tissues (Figure 1B). Our unpublished data further suggested that the larger sizes of the bands were cytoplasm specific, and aging decreased the cytoplasmic expression of TET2. These data implicated that the punctuated signals of TET2 around nucleus might represent mitochondrial distribution, and cytoplasm-localized TET2 might participate in neuronal development.

One study investigated the mechanism of DMSO-induced cell death; the authors indicated that the increase of death related signals, Fas and Dlx5, were due to up-regulation of TET and 5hmC [22]. Another study showed that TET1-mediated DNA demethylation regulated oxidative stress-induced neuron death, and this function depended on the CD domain, which conversed 5mC to 5hmC [23]. Meanwhile, gene knockout mice also gave us some valuable information. In a study from 2012, most TET2 knockout mice died at postnatal-day three, indicating the close link between TET2 and survival [7]. Controversially, some labs suggested that combined deficiency of TET1 and TET2 caused epigenetic abnormalities, but was compatible with postnatal development [24]. This discrepancy may be due to different gene knockout strategies. The Shide group used Ayu17-449 (*TET2^trap^*) mice in which the full-length of TET2 containing N-terminus and CD domain (Cys-rich and DSBH regions) was totally knocked out, meanwhile, some other studies only knockedout the CD domain of TET2 [25]. Therefore, these seemingly contradictory results highly illustrated that TET2 implemented the survival role independent of its CD domain. One investigation between TET2 and cerebral hypoperfusion suggested that increase of TET2 in the cerebral cortex was essential for neuron survival; however, there is no corresponding increase in 5hmC [26]. Consistently, other studies also strongly suggested that TET exerted its role independent of 5hmC, and, instead, through the complicated interactions with some transcription related proteins, such as *O*-linked β-*N*-acetylglucosamine (*O*-GlcNAc) transferase (OGT) [27]. In our study, we used shRNA to down-regulate TET2 in cultured cortex neurons, and, compared to controls, these cells were more prone to death, strongly indicating that TET2 was necessary for cell survival. In addition, TET1 and TET3 expression level was not influenced in TET2-deficient cells (Figure 3C), thus, they did not compensate sufficiently for the loss-of-function of TET2. Whether the CD domain of TET2 or cytoplasm localized TET2 contributes to this function should be deeply studied.

Our unpublished data suggested that mRNA and protein of TET2 increased notably in brain tissues of transgenetic mice model of Alzheimer’s disease and cerebral ischemic injury mice. Since oxidative stress is the pivotal link in those neurodegenerative diseases [28], we used hippocampal neuron cell lines HT22 to find that knockdown of TET2 led to more cell death when exposed to H_2_O_2_. In the meantime, we also found that most glia cells depleted of TET2 live healthily (Figure 3C), which may be due to the fact that neurons are more sensitive to oxidative stress than glia cells. Thus, neuronal TET2 may regulate redox-related genes’ balance to sustain cell survival, and the possibility should be further checked. Further research should be done to examine the molecular mechanism under these protections. In addition, the cell specific distribution and potential functions of TET1 and TET3 should also be deeply studied.

## 3. Experimental Section

### 3.1. Animals

All animal procedures were approved by the Animal Care and Ethical Committee at Xi’an Medical University (Permit Number: 2012-8, 7 March 2012). All efforts were made to reduce the number of animals used by following the 3Rs (reduction, refinement, and replacement). Embryonic (E) 16–18 day, 1 week and 10 week C57BL/6 mice were obtained from the Experimental Animal Center of Xi’an Jiao Tong University.

### 3.2. Antibodies

Rabbit polyclonal antibody against TET2 was purchased from pTGLAB and this antibody could specifically recognize TET2 protein in immunofluerescence and Western blot [19]. Anti-NeuN antibody, which is commonly used as a specific marker for neurons, was purchased from Millipore (Boston, MA, USA), another neuron marker, TuJ1, was from Sigma-Aldrich (San Francisco, CA, USA). Inner standard α-tubulin was purchased from Santa Cruz Biotechnology (Santa Cruz, CA, USA).

### 3.3. Cell Culture

Primary cortical neurons were prepared from brains of E16–18 day C57BL/6 mice, as described previously [29,30]. Briefly, the cerebral cortex was removed aseptically, then digested and dispersed into single cells. After centrifugation, neurons were resuspended in Dulbecco’s modified Eagle’s medium (DMEM) with 10% fetal bovine serum (FBS), and finally seeded at 1.25 × 10^5^ cells/cm^2^ on culture plates (Corning, Corning, NY, USA) coated with 100 μg/mL poly-l-lysine and incubated in a humidified atmosphere with 5% CO_2_ at 37 °C. Two hours later, the whole medium was replaced with Neurobasal medium containing 2% B27 supplement (Invitrogen, Carlsbad, CA, USA).

### 3.4. TET2 Short Hairpin RNA (shRNAs) and Lentivirus Infection

Two pairs of mouse TET2 shRNAs (GGATGTAAGTTTGCCAGAAGC, named TET2 shRNA-1#, and GGGTAAGCCAAGAAAGAAA, named TET2 shRNA-2#) [2] were constructed, respectively, by Genepharma (Shanghai GenePharma Co., Ltd) into a lentivirus expression vector pGLVH1/GFP+Puro. Then, the lentiviruses carrying the two TET2 shRNAs were packaged, respectively. The efficacy of TET2 knockdown was examined in cortical neurons. Western blot data showed that TET2 shRNA-2# was more highly effective than TET2 shRNA-1# (Figure 3B), and, thus, we selected TET2 shRNA-2# in the following experiments.

For primary cortical neurons, the effective lentivirus-mediated TET2 shRNA-2# was used to infect neurons 2 h after cell plating. Seventy-two hours later, neurons were subjected to cell death assay and MTT assay.

### 3.5. Cell Death Assay

The neurons from different groups were incubated in culture medium containing propidium iodide (PI) and Hoechst at 37 °C for 5 min. PI could mark the Necrotic and the late apoptotic cells, while Hoechst could mark all cells. Thus, percentage of cell death could be calculated by PI (+)/Hoechst (+).

### 3.6. MTT Assay

Cell viability was assessed by measuring their ability to metabolize 3-(4,5-dimethyldiazol-2-yl)-2,5-diphenyltetrazolium bromide (MTT). Following the indicated treatments, cells were maintained in growth medium containing 0.5 mg/mL MTT for 4 h at 37 °C. After removing the medium, DMSO was then added to each individual well. Following complete dissolution for 10 min, absorbance at 490 nm was measured.

### 3.7. Western Blot

The procedure had been previously described [31]. In brief, the total brain tissue or cell lysate extracts were prepared in high KCl lysis buffer with complete protease inhibitor cocktail (Roche, Basel, Switzerland). The samples were separated by SDS-PAGE and transferred to polyvinylidene fluoride membranes (Roche). The membranes were treated with 1% blocking solution in TBS, followed by incubation with primary antibodies and then POD-labeled secondary antibodies (Roche). The immunolabeled proteins were detected by the BM Chemiluminescence Western Blotting kit (Roche). The primary antibodies used were as follows: TET2 (1: 2500), α-tubulin (1:2000). α-Tubulin was chosen as a loading control.

### 3.8. Immunohistochemistry and Immunocytochemistry

The animals were anesthetized by intraperitoneal injection of sodium pentobarbital (45 mg/kg) and perfused transcardially with 0.9% saline followed by ice-cold 4% paraformaldehyde. The brains were removed, postfixed in the same fixative for 2–4 h, and cryoprotected in 30% sucrose at 4 °C for 24 h. Twenty micrometer sections were cut on a cryostat. The sections were treated for 1 h in PBS containing 3% bovine serum albumin and 0.3% Triton X-100 and incubated overnight at 4 °C with the primary antibody against TET2 (1:250) and NeuN (1:1000). Then, the sections were incubated with Alexa Fluor 488 donkey anti-rabbit IgG (1:800, Molecular Probes, New York, NY, USA) and Alexa Fluor 596 donkey anti-mouse IgG (1:800, Molecular Probes). Finally, the sections were stained with DAPI for 5 min and mounted.

Neurons at 3 days *in vitro* (DIV) on poly-l-lysine-coated glass coverslips were fixed with 4% paraformaldehyde for 15 min at room temperature and then permeabilized by treatment with ice-cold methanol for 10 min. After being blocked by 10% normal donkey serum for 30 min, cells were incubated at room temperature for 1 h with primary antibody TET2 (1:250) and TuJ1 (1:1000). Then they were rinsed and incubated for 1 h at room temperature with secondary antibodies and then mounted as above.

The labeled sections and cells were observed under an Olympus Confocal Microscope (FV1000).

### 3.9. RT-PCR

Total RNA from different brain tissues was isolated using Trizol Reagent (Invitrogen) and was subsequently treated with RNase-free DNase I (Roche). Synthesis of cDNA was performed by using a First Strand cDNA Synthesis Kit (TOYOBO, Osaka, Japan) according to the manufacturer’s instructions. Quantitative real-time PCR was performed using ABI Stepone plus and Realtime PCR Master Mix (SYBR Green) (TOYOBO). β-Actin was chosen as the endogenous control in the assay. Primers for real-time PCR of TET2 were 5′-TGTTGTTGTCAGGGTGAGAATC-3′ (forward) and 5′-TCTTGCTTCTGGCAAACTTACA-3′ (reverse). TET1, 5′-GAGCCTGTTCCTCGATGTGG-3′ (forward) and 5′-CAAACCCACCTGAGGCTGTT-3′ (reverse); TET3, 5′-CCGGATTGAGAAGGTCATCTAC-3′ (forward) and 5′-AAGATAACAATCACGGCGTTCT-3′ (reverse).

### 3.10. Statistical Analysis

The data shown in this study were expressed as Mean ± S.D. Statistical significance was evaluated by paired *t*-test, and probability values of less than 5% were considered significant.

## 4. Conclusions

Our data firstly clarified that TET2 was abundantly expressed in most neurons of the cerebral cortex and hippocampus. Further investigations showed that TET2 localized in both the nucleus and cytoplasm of neurons. More importantly, neurons missing TET2 were prone to death. All these data uncovered a novel function of neuronal TET2 that may be essential for autonomous cell survival.
